# Plasma oxalate: comparison of methodologies

**DOI:** 10.1007/s00240-020-01197-4

**Published:** 2020-05-29

**Authors:** Felicity Stokes, Cecile Acquaviva-Bourdain, Bernd Hoppe, John C. Lieske, Elisabeth Lindner, Greg Toulson, Frédéric M. Vaz, Gill Rumsby

**Affiliations:** 1 Manual Blood Sciences, Health Services Laboratories, The Halo Building, 1 Mabledon Place, London, WC1H 9AX UK; 2 Laboratory Biochemistry and Molecular Biology, Inborn Errors of Metabolism Unit, Hospices Civils de Lyon, Centre de Biologie Est, 69500 Bron, France; 3 German Hyperoxaluria Center, University Hospital Bonn, Venusberg Campus 1, 53127 Bonn, Germany; 4 Department of Laboratory Medicine and Pathology, Division of Nephrology and Hypertension, Mayo Clinic, 200 First St SW, Rochester, MN 55905 USA; 5 OxThera AB, Regeringsgatan 111, 111 39 Stockholm, Sweden; 6 Clinical Biochemistry, Birmingham Children’s Hospital, Steelhouse Lane, Birmingham, UK; 7 Laboratory Genetic Metabolic Diseases, Core Facility Metabolomics, Amsterdam UMC, University of Amsterdam, Amsterdam Gastroenterology and Metabolism, Meibergdreef 9, Amsterdam, The Netherlands; 8 Infection and Immunity, University College London, London, UK

**Keywords:** Plasma oxalate, Method comparison, Ultrafiltration, Primary hyperoxaluria

## Abstract

Measurement of oxalate in the blood is essential for monitoring primary hyperoxaluria patients with progressive renal impairment and on dialysis prior to transplantation. As no external quality assurance scheme is available for this analyte, we conducted a sample exchange scheme between six laboratories specifically involved with the investigation of primary hyperoxaluria to compare results. The methodologies compared were gas chromatography/mass spectrometry (GCMS), ion chromatography with mass spectrometry (ICMS), and enzymatic methods using oxalate oxidase and spectrophotometry. Although individual laboratories performed well in terms of reproducibility and linearity, there was poor agreement (absolute values) between centres as illustrated by a longer-term comparison of patient results from two of the participating laboratories. This situation was only partly related to differences in calibration and mainly reflected the lower recoveries seen with the ultrafiltration of samples. These findings lead us to conclude that longitudinal monitoring of primary hyperoxaluria patients with deteriorating kidney function should be performed by a single consistent laboratory and the methodology used should always be defined. In addition, plasma oxalate concentrations reported in registry studies and those associated with the risk of systemic oxalosis in published studies need to be interpreted in light of the methodology used. A reference method and external quality assurance scheme for plasma oxalate analysis would be beneficial.

## Introduction

Oxalate is an end product of human metabolism that is cleared by the kidneys and excreted in the urine. The low solubility of its calcium salt makes it a particular problem when oxalate is present in the urine in excess, as it forms crystals and kidney stones, and in extreme cases can lead to renal damage. Since oxalate is endogenously produced and primarily excreted by the kidneys, in chronic kidney disease blood oxalate concentrations increase. In diseases associated with increased oxalate loads, supersaturation with calcium oxalate and precipitation of crystals can occur in tissues including the eye, bones, heart, and vascular systems (systemic oxalosis).

Disorders associated with excessive oxalate production include ethylene glycol toxicity, enteric hyperoxaluria, and the primary hyperoxalurias (PH). In all cases, plasma oxalate levels remain relatively normal if GFR is preserved. At CKD stage 3 or greater plasma oxalate gradually rises. Thus, plasma oxalate is positively correlated with plasma creatinine [1] and negatively correlated with eGFR [2, 3]. Concentrations of plasma oxalate in PH patients can overlap with those of patients seen in renal failure from any cause [3, 4] and therefore are not always diagnostic for PH. However, in CKD stage 4 or greater, values > 50 µmol/L are often observed in PH and is thus highly suggestive of the diagnosis and warrants further diagnostic testing: urinary PH metabolites (glycolate, glycerate and dihydroxyglutarate [5]) and genetic testing for the 3 known causative genes (*AGXT*, *GRHPR*, *HOGA1*) [6].

Measurement of blood oxalate is challenging due to its micromolar concentration and issues related to non-enzymatic generation in vitro. Thus, pre-analytic and analytic considerations are both important. Published methods before 1988 often describe alkalinization of the sample and prolonged centrifugation through a variety of filters to deproteinize the samples. It has since been established that alkalinization can contribute to artefactual increases in oxalate as a result of the non-enzymatic metabolism of ascorbate that can occur at a pH > 5 [1, 7], while the choice of the filter can greatly influence recovery [8]. Thus, results using these early methods, particularly published reference ranges, are often hard to interpret. Furthermore, a number of analytic methods have been described including enzymatic oxalate oxidase [9], gas chromatography (GC) [10, 11] with or without mass spectrometry (MS), liquid chromatography with and without mass spectrometry [12–14] and ion chromatography with or without MS detection (ICMS) [8, 15].

To date, there has been no publication that extensively and directly compares methods that are currently used to monitor PH patients with impaired renal function and/or on dialysis, and as endpoints for clinical trials in PH. Thus, the aim of this manuscript is to compare results of blood oxalate analysis using several methods currently employed in clinical laboratories actively engaged in monitoring PH patients. The significance of these results for the interpretation of clinical data is discussed in the setting of newer clinical trial data.

## Methods

### Clinical samples

Longitudinal blood samples collected into lithium heparin were taken from 8 PH patients over several weeks (range 14–156) as part of an ongoing 36-month clinical study treating anuric PH-patients in end-stage renal disease (Oxabact OC5-OL01, OxThera, Sweden). The study used a standardized procedure with a morning sampling taken from each patient once a month prior to the third dialysis day during the week. As per protocol, plasma oxalate was analysed by both ICMS and GCMS. The clinical protocol and the current ancillary study were approved by the University of Bonn Faculty of Medicine; Ethics Committee, Sud-Est II, Independent Ethics Committee, Lyon; and Mayo Clinic Institutional Review Board, Rochester Minnesota, USA.

### Method comparison

Inter-laboratory method comparisons were conducted in 3 stages over 18 months. Analytical methods employed by the participating laboratories were GCMS (*n* = 2), enzymatic oxalate oxidase with colorimetric detection (*n* = 3), and ICMS (*n* = 1). All samples were sent out blinded.

In stage 1, time-expired, citrated fresh frozen plasma was obtained from the blood bank to provide sufficient medium. This material was distributed neatly and spiked with 1000 µmol/L oxalate standard solution (Trinity Biotech, Ireland) to increase the concentration by 11 µmol/L and 35 µmol/L respectively. Samples were sent out as blinded duplicates to assess individual method repeatability and to allow comparison between methods.

In the 2nd stage, an EDTA plasma sample from a patient with a raised plasma oxalate concentration was used to determine whether this sample behaved differently from the in vitro spiked plasma used in stage 1. To assess linearity, serial dilutions (1 in 2 and 1 in 4) of this sample were performed using a low oxalate EDTA plasma sample to avoid matrix changes. This baseline (diluent) sample was also distributed for analysis. In this stage samples were sent out blinded in singlicate.

In both stages 1 and 2, all samples were frozen (− 20 °C) prior to shipment on dry ice. At the receiving laboratories, samples were kept frozen until analysis and all samples in a shipment were assayed as a single batch as quickly as possible according to the standard protocol of each laboratory (Table 1).

**Table 1:** Sample preparation steps for the individual laboratories

Laboratory	Dilution/diluent	Filter (cut off)	Centrifugation	Extraction	Method (platform)	Stated linearity (µmol/L)
1	1:1 with 0.24 M HCl prior to filtration	Amicon ultra (30 kDa)	18,000 g 4 °C, 10 min	No	Oxalate oxidase (Ilab Aries)	2–60 (up to 200 on dilution)
2	1:1 with water prior to filtration	Amicon ultra (30 kDa)	15,000 g 4 °C, 10 min	No	Oxalate oxidase (Indiko Plus)	4–200
3	1:1 with NaCl + 200 µL 6 M HCl	None		Ethyl acetate	GCMS	2–60
4	200 μL + 30 μL 12 M HCl	None		Ethyl acetate	GCMS	0.5–420
5	500 μL + 20 μL 2 M HCl (added to filtrate after ultrafiltration)	Centrisart I ultrafiltration vial (10 kDa)	1500 g 4 °C, 20 min		ICMS	0.3-upper limit not defined
6	1 mL plasma + 10 μL 12 M HCl	Amicon ultra (30 kDa)	2300 g, 20 °C, 30 min		Oxalate oxidase (microwell plate)	1–300

In stage 3, a 1000 µmol/L aqueous oxalate standard solution was diluted with deionised water to produce final concentrations of 150, 50, and 25 µmol/L. These solutions were sent out as blinded duplicates to each laboratory for direct analysis without any additional sample processing. This comparison allowed assessment of calibration and method related bias by removing any pre-analytical variation. This was the only stage where samples were given an assigned value.

### Sample preparation

The participating laboratories used their own sample preparation methodology, as described in Table 1.

With the exception of laboratories 3 and 4, all laboratories used ultrafiltration during sample preparation to remove protein either without acidification (lab 2), with acidification prior to ultra-filtration (lab 1 and 6) or acidification after ultrafiltration (lab 5). Lab 3 and 4 used acidification plus the extraction of oxalate.

### Statistical analysis

Mean and standard deviation was calculated for blinded duplicate samples for each laboratory to assess intra-assay variation. The mean and coefficient of variation were calculated for each sample to assess inter-laboratory variability. All statistical analyses and Bland–Altman plots [16] were determined using Excel.

## Results

### Clinical samples

**Fig. 1:**
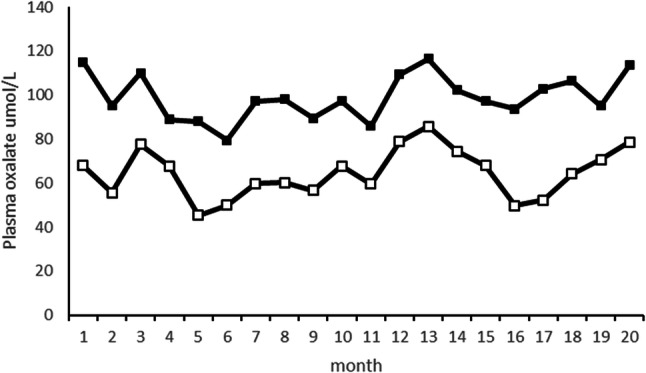
Comparison of plasma oxalate results obtained from a PH patient over a period of months. Solid symbols (filled square) GCMS, open (unfilled square) ICMS results

**Fig. 2:**
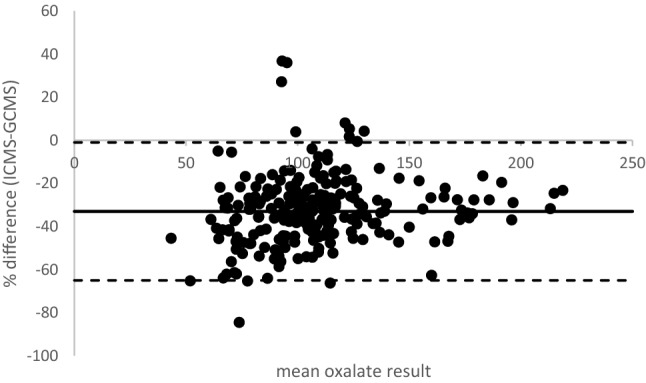
Bias plot of results obtained by ICMS and GCMS. The solid line denotes mean % difference; dashed lines denote ± 2SD

As part of the ongoing clinical study, plasma oxalate was measured by both GCMS and ICMS in 233 samples from 8 PH patients over various time periods as detailed in the “Methods” section. Although the results were highly correlated, as illustrated by the patient depicted in Fig. 1, there was a significant bias between the 2 methods with ICMS results substantially lower with an overall mean of 73% compared to GCMS (range 64–79%). A Bland–Altman plot [16] of 233 results confirmed that ICMS had a − 33% negative bias when compared to GCMS (Fig. 2). This observation prompted the current ancillary protocol to better understand the extent and nature of variability in plasma oxalate between assays as currently analysed in clinical laboratories.

## Method comparison

### Stage 1

There was poor agreement between laboratories with respect to the baseline sample (Table 2) with values ranging from 6 to 15 μmol/L, although reproducibility for individual methods was good and recoveries were typically > 90% with the exception of ICMS (laboratory 5) and one of the oxalate oxidase assays (laboratory 6), where recoveries were below 80% (Table 3).

**Table 2:** Results from spiked samples

Laboratory	Mean result (SD)		
	Neat plasma	+ 11 μmol/L	+ 35 μmol/L
1	7 (0.2)	17 (0.5)	39 (0.2)
2	13 (0.3)	23 (0.1)	47 (0.6)
3	6 (1.0)	18 (0.7)	47 (1.4)
4	9 (0.2)	21 (0.1)	47 (0.6)
5	15 (0.3)	24 (0.5)	45 (0.8)
6	6 (0.2)	14 (0.1)	33 (0.3)
All lab mean (%CV)	9 (41.6)	19.3 (17.7)	43.2 (12.7)

Oxalate results are reported in whole numbers, standard deviation to 1 dp. SD were calculated for the blinded duplicate samples for each lab. Inter-laboratory CV was calculated for each sample

*CV* coefficient of variation

**Table 3:** Recovery from spiked samples

Laboratory	% Recovery	
	+ 11 μmol/L	+ 35 μmol/L
1	90	91
2	92	98
3	105	117
4	105	108
5	77	84
6	77	77

Recovery = (spiked result-neat plasma)/amount spiked)

### Stage 2

In this stage a blood sample from a PH patient with a high plasma oxalate concentration was used to assess the dilution linearity and assay comparison of endogenous oxalate.

**Fig. 3:**
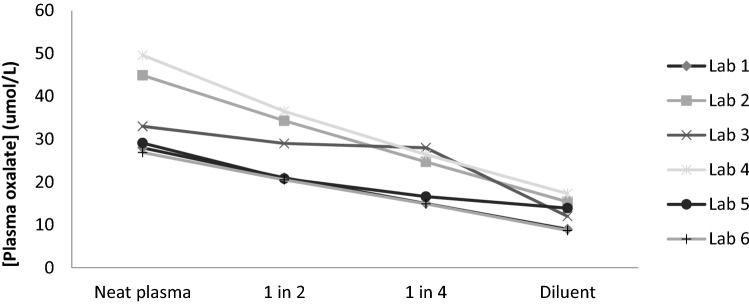
Results obtained following dilution of human plasma

As Table 4 and Fig. 3 demonstrate the majority of methods produced a linear response to dilution, with the exception of laboratory 3. No explanation was apparent and there was insufficient material to reanalyse. There was a significant range of results for the PH patient plasma, ranging from 27 to 50 μmol/L, with 2 of the 3 oxalate oxidase assays giving the lowest results. Lab 1, 2, and 6 use enzymatic assays. Of note, is that Lab 2, where the sample was not pre-acidified showed a high value, while Lab 1 and 6 also using enzymatic methodology had the lowest results. Lab 5 uses post-ultrafilter acidification with a smaller pore filter and ICMS analysis, but also showed a low value. No conclusion can, therefore, be drawn regarding the use of acidification. In all cases these results were within the stated reporting range for the respective methods (Table 1).

**Table 4:** Analysis of a high oxalate blood sample diluted with plasma to minimise the change in matrix

Laboratory	Plasma oxalate result (μmol/L)			
	PH patient plasma	1 in 2	1 in 4	Diluent plasma
1	28	21	15	9
2	45	34	25	15
3	33	29	28	12
4	50	37	26	17
5	29	21	17	14
6	27	21	15	9
All lab mean (%CV)	36 (25.1)	28 (24.8)	21 (26.4)	13 (25.0)

### Stage 3

**Fig. 4:**
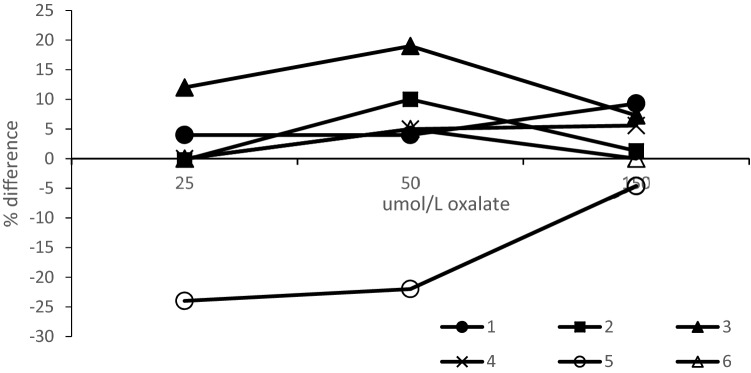
Comparison of aqueous standards by the six different laboratories. The % difference is plotted against the known oxalate concentration

In stage 3 aqueous oxalate standards were sent out to the participating laboratories in order to determine whether differences between the methods were related to calibration, rather than assay dependent variation or pre-analytic steps in preparation of samples in patient matrix. In most cases the linearity of response was good but two of the methods (laboratories 3 and 5) demonstrated a tendency to a positive and negative bias respectively with the 25 and 50 μmol/L aqueous standards (Table 5). A difference plot was prepared to allow a visual comparison of the results with the amount of aqueous standard on the *x*-axis rather than the grand mean since the true value of the standard material is known (Fig. 4).

**Table 5:** Results from analysis of duplicate aqueous standards

Laboratory	Added oxalate (µmol/L)		
	150	50	25
	Mean measured oxalate μmol/L (SD)		
1	164 (1.4)	52 (0)	26 (0)
2	152 (2.0)	50 (0.1)	25 (0.6)
3	161 (4.2)	60 (0.8)	28 (0)
4	158 (0.6)	52 (0.7)	25 (0.1)
5	143 (0)	39 (0.3)	19 (0)
6	150 (0.6)	53 (3.5)	25 (0.2)
All lab mean (%CV)	155 (4.9)	51 (12.2)	25 (12.1)

Oxalate results are reported in whole numbers, standard deviation to 1 dp. SD was calculated for the blinded duplicate samples for each lab. Inter-laboratory CV was calculated for each sample

*CV* coefficient of variation

The majority of the methods gave acceptable results for this part of the study suggesting that calibration is not a major issue in most cases although it should be reviewed by laboratories 3 and 5.

**Fig. 5:**
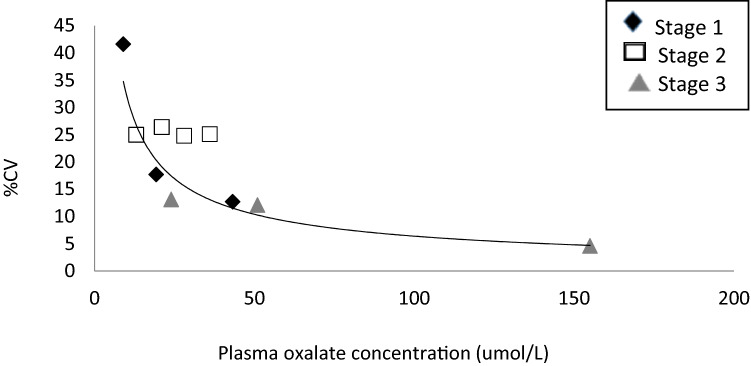
Precision plot of inter-laboratory %CVs from each stage of the method comparison

The inter-laboratory % coefficient of variation (%CV) was also calculated for the samples distributed in each stage (Fig. 5). While the variation between laboratories in stages 1 and 3 (spiked oxalate) follow a similar precision profile with a decrease in %CV with concentration as would be expected, there is a higher variation of submitted results between laboratories in stage 2 (endogenous oxalate). This finding raises the question of whether the analysis of endogenous oxalate is subject to increased pre-analytic or analytic variation.

Overall the results show that there is poor agreement between methods, particularly at the low end i.e. within the reference range. The lower patient results obtained with ICMS can be partially explained by differences in recovery from the ultrafiltrate for this particular method that uses a smaller pore ultrafiltration device (Table 1). However, as some negative bias is still present for this method following direct analysis of the aqueous sample in stage 3, other factors, such as calibration differences, may also play a role. It does not however, explain the whole of the − 33% bias of ICMS method versus GCMS.

For the enzymatic assays, it seems that the pre-acidification of samples gives a lower result (results were not negatively biased in stage 3 where ultrafiltration did not take place). While it is possible that the acidification may be denaturing protein and blocking the ultrafilter leading to lower recoveries, there is no evidence for this from the stage 1 method comparison. As an extension to this study a comparison of recoveries for individual enzymatic methods using pre-acidification and no acidification may be beneficial.

## Discussion

Measurement of the oxalate content of blood is useful in monitoring patients with PH, particularly those with impaired renal function. It is complicated by both sample instability and by differences in sample preparation, potentially leading to the impaired recovery of oxalate and differences in results. In the absence of matrix-matched certified reference material and a recognised external quality assurance scheme, sample exchange is the only means available to compare assays. With the consideration of this analyte as an endpoint in clinical trials for PH treatment, it seemed timely to conduct a comparison of results across laboratories. We have interpreted the data from the participating laboratories using the guidelines set out by European Accreditation EA-4/21 INF:2018 [17]. One limitation of the present study was the need to send out the same sample type to all laboratories. In some cases, this material was a deviation from the normal sample requirement (such as serum or heparinised plasma) for a particular laboratory. Other limitations are the small sample size necessitated by the high volume of sample required and the possibility that not all oxalate methodologies have been included in the study.

A comparison of patients’ samples analysed by GCMS and ICMS showed that the results mirrored each other but with significant bias between the methods. In seeking a cause for this bias, we formally investigated the contribution of variation in pre-analytical and analytical recovery through the method comparison studies.

There was poor agreement at the low end of results for the unspiked plasma samples distributed in stages 1 and 2, range 6–15 μmol/L and 9–17 μmol/L respectively. Possible causes for such variation may be non-specific spectral interferences in the spectrophotometric assays, matrix effects in the chromatography methods, differences in recovery during ultrafiltration, calibration differences, or inadequate blanking. Oxalate contamination of filters has been reported, particularly for the Centrisart 1 used by one laboratory [8] but would be unlikely to contribute more than 1–2 μmol/L. Ascorbate is known to be converted to oxalate in vitro and can lead to increased results [1, 18]. Care was taken to minimise this factor by freezing samples immediately after preparation and requesting analysis promptly following receipt, but its contribution cannot be completely excluded.

There is some suggestion from the imprecision plot (Fig. 5) that endogenous oxalate in plasma may behave differently than spiked material. The oxalate oxidase and ICMS methods require deproteinization of the plasma sample by ultrafiltration, a procedure that is known to lead to reduced overall recoveries with wide variation among filtration devices [8]. In addition, aqueous standards that are not subject to the ultrafiltration step are used in some assays, which means this loss is uncompensated. Losses, determined by 14C labelling, occur by binding of oxalate to the filter [8] and to plasma protein [19] and one question we had at the initiation of this study was whether the latter led to the discrepancy in patient results between methods. Protein binding is pH-dependent with good recoveries seen at physiological pH and at less than pH 2 but dropping to 30–40% at intermediate pH [18, 20, 21]. In the majority of cases described in the current study, recoveries were more than 80% and greater than 100% with GCMS. Where ultrafiltration was used, it was performed either with pre-acidification to a low pH or at neutral pH with or without later acidification. GCMS, by contrast, uses an extraction technique and is therefore not subject to the same losses, although protein precipitation is performed prior to extraction.

The lowest inter-laboratory variation was observed when aqueous samples were distributed to be analysed directly with very good agreement between most laboratories, indicating that sample preparation, including ultrafiltration, is a major factor involved in inter-laboratory variation. Matrix effects would also have been minimised, and therefore may also contribute to the variation. However, this cannot entirely explain the − 33% bias seen between GCMS and ICMS (Fig. 2), suggesting that other factors, such as calibration may also be contributing and in this regard, we suggest that laboratories 3 and 5 review their choice of calibrators. It should be noted that the concentration of oxalate in the clinical samples was much higher than we were able to provide for the sample exchange and this may have a bearing on the results. Systemic oxalosis occurs at high plasma oxalate levels including deposition in bone marrow [22] and it is possible that oxalate crystals are present in the plasma that would be retained by ultrafiltration exacerbating the difference.

In conclusion, the implications of this study are threefold. Firstly, there are marked differences in results obtained using different methods and therefore longitudinal studies on patients must be carried out using the same laboratory and methodology. In practice, this situation is likely to occur anyway as plasma oxalate tends to be offered only by specialist laboratories but is particularly pertinent for clinical trial samples.

Secondly, published data on the level of plasma oxalate associated with increased risk of supersaturation and therefore of systemic oxalosis [2, 23] will depend on the assay used and thus has implications for clinical target setting and for evaluation of patient registry data. In this case, it would be important to acknowledge the methodology used.

Finally, this study has highlighted the high degree of variation between methods, which is important to address. This situation is not uncommon for analytes where a range of ‘in-house’ methods are used and where sample processing plays an important role. The identification of a definitive method for plasma oxalate, along with a matrix-matched standard reference material would be the first step in improving the situation. A quality assurance scheme would also be required, along with the co-operation of laboratories performing this analysis to be open to future alterations in calibrators and pre-analytic procedures to standardise methodology.
